# Airway Management in A Case of Tongue Flap Division Surgery: A Case Report

**Published:** 2009-02

**Authors:** Tapas Kumar Sahoo, Manasi Ambardekar, R D Patel, S H Pandya

**Affiliations:** 1 Department of Anaesthesiology, Seth G.S. Medical College and K.E.M. Hospital, Mumbai, India; 2 Department of Anaesthesiology, Seth G.S. Medical College and K.E.M. Hospital, Mumbai, India; 3 Department of Anaesthesiology, Seth G.S. Medical College and K.E.M. Hospital, Mumbai, India; 4 Department of Anaesthesiology, Seth G.S. Medical College and K.E.M. Hospital, Mumbai, India

**Keywords:** Tongue flap, Right molar approach, Miller straight blade

## Abstract

**Summary:**

This article sums up successful airway management in an 18-year-old male presented for tongue flap division surgery constructed before for a palatal fistula in our hospital. After induction of general anaesthesia, we performed laryngoscopy with right molar approach using miller straight blade, intubated from right side of flap and throat packing done using left molar approach. Tongue flap was divided without any ties and hemostasis checked.

## Introduction

Tongue flaps are an accepted method of treating defects of the palate. The tongue flap technique is based on the use of a flap constructed from the dorsum of the tongue to close a defect in the palate^1^. These airways are readily managed for the initial flap construction surgery using nasal intubation^2^. Securing the airway for the tongue flap division surgery is more challenging. We report successful airway management for tongue flap division surgery who was previously operated for palatal fistula.

## Case report

**Fig 1:**
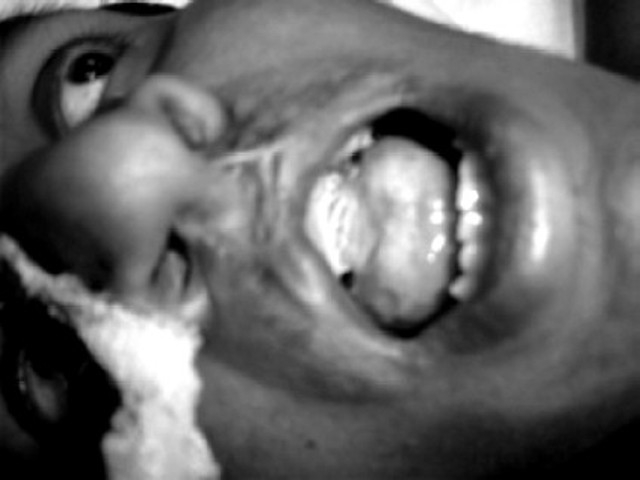
Photograph of the patient showing tongue flap connecting the dorsum of the tongue and the palate

An 18-year-old and 58kg weight male presented for division of tongue flap (Fig 1) constructed 3 weeks ago for a palatal fistula. Past surgical history was significant for bilateral cleft lip and palate repair in childhood and maxillary distraction after LeFort osteotomy 1year back. Past medical history was unremarkable.

All the routine investigations like hematologic, chest x-ray and ECG were normal. On examination of the airway, mouth opening was restricted to 3 finger breadths due to the tongue flap. Uvula and posterior pharyngeal wall couldn't be visualized.

**Fig 2:**
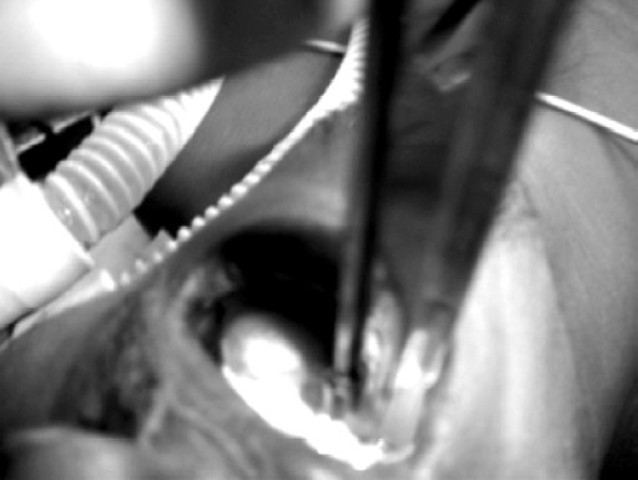
Photograph showing laryngoscopy being done with right molar approach using Miller no.3 straight blade

**Fig 3:**
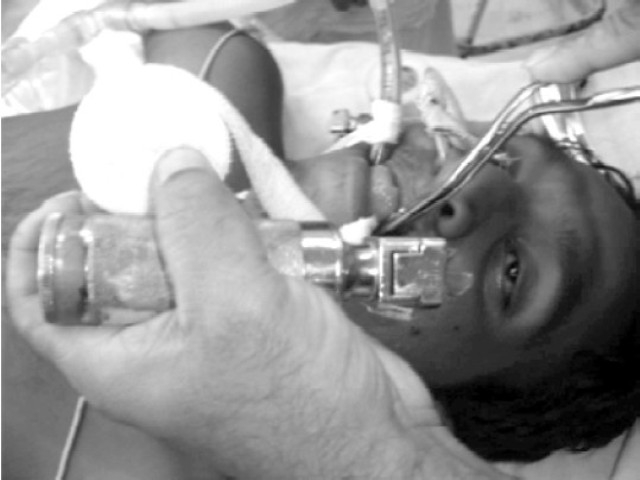
Photograph showing throat packing being done with left molar approach after orotracheal intubation done with right molar approach

On the day of surgery, after confirming adequate starvation and informed consent, patient was taken inside the operation theatre and monitors-cardioscope, pulse oximeter, non-invasive blood pressure cuff and capnometer were attached. Anaesthesia was induced with propofol 120mg i.v. After confirming adequate mask ventilation, pancuronium 4mg was given. Midazolam 1.5mg and pentazocine 18mg i.v. were given for sedation and analgesia. Then both lungs were ventilated with O_2_ and N_2_O (50:50) in a close circuit for 3 minutes. Laryngoscopy was performed with right molar approach using Miller no.3 blade(Fig 2) and trachea was intubated using no.9 PVC cuffed endotracheal tube from right side of flap. Correct placement of tracheal tube was confirmed by bilateral chest auscultation and capnography. Throat packing was done using left molar approach (Fig 3). Flap was divided without any ties and surgery accomplished. Both ends were sutured and hemostasis confirmed. Throughout the surgery, patient's vitals remained within normal range. After completion of surgery, neuromuscular blockade was reversed with neostigmine 3mg and glycopyrrolate 0.4mg i.v. Before extubation, both tongue and palatal sites of attachment were sprayed with one puff each of 10% lidocaine. Trachea was extubated smoothly and patient was observed for 10 minutes before shifting to postoperative recovery room.

## Discussion

Tongue flaps are an accepted method of treating palatal defects. Tongue flap surgery for cleft palate repair involves two separate operations. In the first, a tongue flap is created to close the palatal defect^1^ and in the second, the flap is divided, freeing the tongue from palate. Airway management for the second operation is complicated by the flap between the tongue and the palate.

Tongue flap may be divided under local anaesthesia followed by induction of general anaesthesia. In their letter to the editor, Sherry Peter et al^3^ have suggested division of tongue flap under local anaesthesia without vasoconstrictors before general anaesthesia. They tied two silk threads towards the tongue end of the flap and flap is divided between them. This technique prevents bleeding. If bleeding occurs, it is immediately cauterized with bipolar cautery. After the flap was divided, they proceeded with conventional induction of general anaesthesia and orotracheal intubation.

Julio Hochberg et al^4^ accomplished inhalational induction with 60% N_2_O, 40% O_2_ and 2-2.5% halothane. Red rubber catheters were introduced through both nostrils to the pharynx as airways to facilitate spontaneous ventilation with a high flow of gases. They also ligated the base of the tongue pedicle doubly, using heavy silk. The pedicle was then cut between two ties.

However dividing the flap under local anaesthesia requires patient's cooperation. There may be bleeding into the airway. To avoid the problems, we decided to secure the airway before the division of flap to preclude the possibility of bleeding and aspiration into an unsecured airway.

For the flap division procedure in one patient, Naveen Eipe et al^5^ used ketamine 50 mg i.v. for sedation, glycopyrrolate 0.2 mg i.v. to control secretions. The surgeons inserted a mouth gag and proceeded to divide the flap with the patient breathing spontaneously. After successful flap division, the patient was anaesthetized, paralyzed, and orotracheal intubation was performed under laryngoscopic visualization without incident.

In the second patient, they used intravenous ketamine to induce general anaesthesia followed by ventilation with O_2_: N_2_O (50:50) and halothane 1% inhalation. They performed an initial laryngoscopy with the head turned to right and laryngoscope blade carefully inserted to the left of the flap using retromolar approach. After confirming laryngeal visualization, intravenous succinylcholine 50mg was administered and direct laryngoscopy performed. An orotracheal tube was passed to the left of the flap. Here it should be emphasized that after any palatoplasty, it is advisable to avoid nasal intubation as this may damage or disrupt the recently constructed flap^6^.

The molar approach reduces the distance from the patient's teeth to the larynx and prevents intrusion of maxillary structures into the line of view. A right molar approach has an additional advantage that the bulging of tongue over the blade is prevented unlike the midline approach.

Henderson^7^ has described use of paraglossal approach using a straight blade for patients with difficult airway. However Ken Yamamoto et al^8^ observed that the left molar approach using a standard Macintosh blade improved the laryngoscopic view in patients with difficult midline laryngoscopy. Ken Yamamoto et al^8^ compared direct laryngoscopic view using midline, left and right molar approaches with Macintosh no.3 or no.4 blade in patients undergoing general anaesthesia for elective surgery.

**Fig 4:**
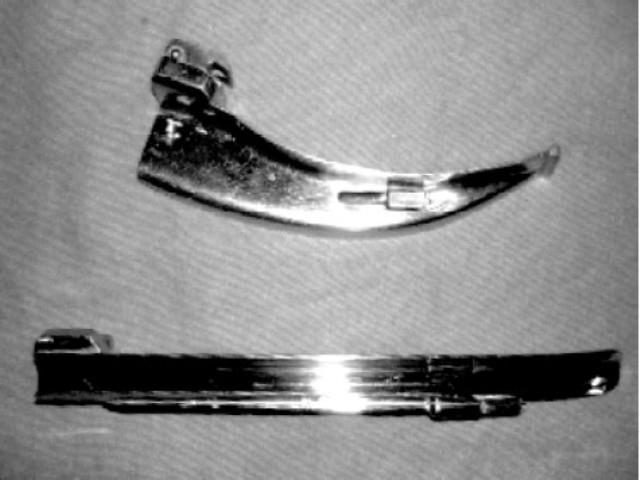
Photograph showing comparison between Macintosh no.3 curved blade and Miller no.3 straight blade

In our case, we decided to usea right molar approach with a straight blade. The breadth of straight Miller no.3 blade is lesser than the curved Macintosh no.3 blade(Fig 4). This minimized the trauma to the tongue flap and provided adequate space for intubation. The drawback of left molar approach by causing bulging of the tongue over the blade and obscuring the glottic view was overcome by using right molar approach. We chose to induce general anaesthesia before division of the flap for airway protection and for patient, surgeons and anaesthesiologists' comfort. Laryngoscopy for packing the throat was done using left molar approach as the endotracheal tube occupied right sided space.

In our opinion, any approach can beused though Miller straight blade to be preferred over Macintosh curved blade in tongue flap division surgery for securing airway. The choice depends on anaesthesiologists' expertise. However, orotracheal intubation using a fibreoptic scope remains the preferred option in such cases. But it may not be available freely in developing countries like India. So familiarity with use of different laryngoscopic blades and approaches is essential for the anaesthesiologists.
